# Type-Specific Cervico-Vaginal Human Papillomavirus Infection Increases Risk of HIV Acquisition Independent of Other Sexually Transmitted Infections

**DOI:** 10.1371/journal.pone.0010094

**Published:** 2010-04-08

**Authors:** Karen K. Smith-McCune, Stephen Shiboski, Mike Z. Chirenje, Tsitsi Magure, Jennifer Tuveson, Yifei Ma, Maria Da Costa, Anna-Barbara Moscicki, Joel M. Palefsky, Rudo Makunike-Mutasa, Tsungai Chipato, Ariane van der Straten, George F. Sawaya

**Affiliations:** 1 Department of Obstetrics, Gynecology and Reproductive Sciences, University of California San Francisco, San Francisco, California, United States of America; 2 Helen Diller Family Comprehensive Cancer Center, University of California San Francisco, San Francisco, California, United States of America; 3 Department of Epidemiology and Biostatistics, University of California San Francisco, San Francisco, California, United States of America; 4 Department of Pediatrics, University of California San Francisco, San Francisco, California, United States of America; 5 Department of Medicine, University of California San Francisco, San Francisco, California, United States of America; 6 Department of Obstetrics and Gynecology, University of Zimbabwe, Harare, Zimbabwe; 7 Women's Global Health Imperative, RTI International, San Francisco, California, United States of America; Louisiana State University, United States of America

## Abstract

**Background:**

Sexually transmitted infections (STIs) such as herpes simplex virus (HSV)-2 are associated with an increased risk of HIV infection. Human papillomavirus (HPV) is a common STI, but little is know about its role in HIV transmission. The objective of this study was to determine whether cervico-vaginal HPV infection increases the risk of HIV acquisition in women independent of other common STIs.

**Methods and Findings:**

This prospective cohort study followed 2040 HIV-negative Zimbabwean women (average age 27 years, range 18–49 years) for a median of 21 months. Participants were tested quarterly for 29 HPV types (with L1 PCR primers) and HIV (antibody testing on blood samples with DNA or RNA PCR confirmation). HIV incidence was 2.7 per 100 woman-years. Baseline HPV prevalence was 24.5%, and the most prevalent HPV types were 58 (5.0%), 16 (4.7%), 70 (2.4%), and 18 (2.3%). In separate regression models adjusting for baseline variables (including age, high risk partner, positive test for STIs, positive HSV-2 serology and condom use), HIV acquisition was associated with having baseline prevalent infection with HPV 58 (aHR 2.13; 95% CI 1.09–4.15) or HPV 70 (aHR 2.68; 95% CI 1.08–6.66). In separate regression models adjusting for both baseline variables and time-dependent variables (including HSV-2 status, incident STIs, new sexual partner and condom use), HIV acquisition was associated with concurrent infection with any non-oncogenic HPV type (aHR 1.70; 95% CI 1.02–2.85), any oncogenic HPV type (aHR 1.96; 95% CI 1.16–3.30), HPV 31 (aHR 4.25; 95% CI 1.81–9.97) or HPV 70 (aHR 3.30; 95% CI 1.50–7.20). Detection of any oncogenic HPV type within the previous 6 months was an independent predictor of HIV acquisition, regardless of whether HPV status at the HIV acquisition visit was included (aHR 1.95; 95% CI 1.19–3.21) or excluded (aHR 1.96; 95% CI 1.02–2.85) from the analysis.

**Conclusions/Significance:**

Cervico-vaginal HPV infection was associated with an increased risk of HIV acquisition in women, and specific HPV types were implicated in this association. The observational nature of our study precludes establishment of causation between HPV infection and HIV acquisition. However, given the high prevalence of HPV infection in women, further investigation of the role of HPV in HIV transmission is warranted.

## Introduction

Human papillomavirus (HPV) is a common sexually transmitted infection (STI) and has been implicated as a causative agent of anogenital cancers including cervical, vaginal, vulvar, and anal cancer [1], [2]. Over 30 types of HPV can infect the anogenital epithelium, but only 18 types, designated as “oncogenic” types, are implicated in the development of cancer [2]. HPV 16 and HPV 18 are the most common oncogenic types associated with cancer, and are targeted by recently developed vaccines [3], [4], [5]. The “non-oncogenic” HPV types are associated with hyperplastic lesions such as genital warts.

Immune-compromised individuals such as those with HIV suffer from higher rates of HPV infection and disease, higher rates of relapse after treatment of HPV-related diseases, and accelerated development of HPV-associated cancer. Little is known, however, about the converse relationship, i.e. the effect of HPV infection on risk of HIV acquisition. Several viral and bacterial STIs, both ulcerative and non-ulcerative, have been implicated as risk factors for HIV acquisition including syphilis, herpes simplex virus (HSV)-2 [6], [7], [8], *Neisseria gonorrhoeae*, *Chlamydia trachomatis*, and *Trichomonas vaginalis* [9], [10], [11]. Having anal infection with 2 or more HPV types was recently shown to be an independent predictor of HIV seroconversion in a prospective cohort study of men who have sex with men [12]. The aim of this study was to determine the association between HPV infection and HIV acquisition in women in the setting of an effectiveness trial of the diaphragm and lubricant gel for HIV/STI prevention in Zimbabwe.

## Methods

### Study design and participants

This study was performed as a substudy within a randomized trial studying the effect of latex diaphragm and lubricant gel provision on HIV acquisition in the setting of counseling and provision of male condoms in Zimbabwe (the Methods for Improving Reproductive Health in Africa [MIRA] trial); details about the MIRA study has been reported elsewhere (ClinicalTrials.gov number NCT00211459) [13]. Results from this trial demonstrated that diaphragm/gel provision added no significant protection against acquisition of HIV [13], or cervical STIs [14], hence data from the intervention and control arms were pooled for this prospective cohort study. Enrollment into the substudy, as for the parent study, was staggered over a one year period, with the first cohort of enrolled participants followed for 24 months and the last enrolled followed for 12 months; all participants were scheduled for follow-up visits every 3 months. Participants in this prospective cohort study were 2040 HIV-negative women enrolled in MIRA at the Harare, Zimbabwe site after February 2004 who agreed to participate in the HPV sub-study. Assessment of the effect of cervico-vaginal HPV infection on risk of HIV acquisition was a pre-specified aim of the HPV substudy. The provision of diaphragm/gel in the setting of condom provision and counseling had minimal overall effect on HPV incidence or clearance [15]. The study protocol was reviewed and approved by the University of California San Francisco (UCSF) Committee on Human Research, and the Medical Research Council of Zimbabwe, and the Medicines Control Authority of Zimbabwe.

### Study Procedures

At baseline (the screening or enrollment visits), a questionnaire on demographics and sexual behavior was administered, and participants were tested for serologic evidence of HIV and herpes simplex virus (HSV)-2; a blood sample was obtained for syphilis testing, and a urine specimen was obtained for PCR testing for *Neisseria gonorrhoeae*, *Chlamydia trachomatis* and *Trichomonas vaginalis* using methods described elsewhere [13]. A pelvic exam was performed, cervical cytology was obtained, and a cervical sample for HPV testing obtained. At quarterly visits from February 2004 to September 2006, participants were asked about recent medical history and sexual behaviors, and samples were collected for HIV, HSV-2, *Neisseria gonorrhoeae*, *Chlamydia trachomatis*, *Trichomonas vaginalis*, and HPV testing.

Women testing positive for a curable STI were treated when the positive test result was reported. Cervical cytology was interpreted locally in Zimbabwe (R.M.) and reported using the Bethesda system [16]. Any woman with a result of high grade squamous intraepithelial neoplasia, adenocarcinoma in situ or cancer at enrollment was referred for colposcopy; women with low grade squamous intraepithelial neoplasia or atypical squamous cells of undetermined significance underwent repeat cytology testing in 6 months and were referred for colposcopy if that test was abnormal. Women with colposcopically directed biopsies and/or endocervical curettage showing cervical intraepithelial neoplasia 2 or 3 or adenocarcinoma in situ underwent diagnostic excisional procedures by cone biopsy or loop excision.

### Methods for HPV sample collection and testing

Cervical swabs for HPV DNA were collected by clinicians under direct visualization of the cervix at the enrollment, 12-month and exit visits, as previously described [15]. Self-collected vaginal swabs were obtained at 3-month intervals after the enrollment visit as previously described [15]. At the 12-month visit, both clinician- and self-collected samples were collected. HPV testing was performed using MY09/MY11 consensus HPV L1 primers as well as primers for amplification of the human beta-globin gene [15]. Samples that were negative for beta-globin were considered unevaluable. Specimens that tested positive for the consensus HPV sequence were further tested with probes specific to 29 different HPV types (6, 11, 16, 18, 26, 31, 32, 33, 35, 39, 40, 45, 51, 52, 53, 54, 55, 56, 58, 59, 61, 66, 68, 69, 70, 73, 83, 84, 82v), and probes for 10 HPV types in a mixture defined as “mix” (HPV 2, 13, 34, 42, 57, 62, 64, 67, 72, and 82). Of note, HPV 55 is now considered to be a subtype of HPV 44 [17], but HPV 44 was not included in the PCR probes used in this study. Specimens that tested positive for the HPV consensus sequence, but negative for the 39 specific types were described as having “untyped” HPV. DNA samples are processed in a room kept clean of amplified DNA. Each PCR experiment was performed with HPV-negative and HPV-positive controls. In addition, 7 percent of all samples are randomly subjected to a repeat PCR test to determine the reproducibility of the results.

### Methods for HIV testing

At screening and quarterly, two HIV rapid tests were performed on finger-prick or venipuncture blood samples using Determine HIV-1/2 (Abbott Laboratories, Tokyo, Japan) and Oraquick (OraSure technologies, Bethlehem, PA, USA). Discordant results of rapid tests were confirmed on serum samples by ELISA (Vironostika, Biomirieux, Durham NC, USA). Timing of HIV acquisition was assessed by RNA or DNA PCR testing on Dry Blot Spot or serum samples from that visit and from prior visits until a negative PCR test was obtained. The HIV acquisition visit was defined as the visit at which the first positive HIV PCR test was detected.

### Statistical methods

Analyses were performed to look for associations between HIV acquisition and HPV infection following an *a priori* analytic plan. In addition to evaluating potential associations with any HPV type, separate analyses for non-oncogenic and oncogenic HPV types were also performed, given that oncogenic and non-oncogenic HPV types have different biologic behaviors as reflected by rates of persistence [18] and neoplastic potential [2]; these analyses were not mutually exclusive and a participant could contribute to both categories if infected with both oncogenic and non-oncogenic HPV types. Analyses of specific HPV types (those with prevalence ≥1%) were also performed. For these analyses, “mix” and untyped infections were excluded. Because of the multiplicity of individual types considered, we used a conservative Bonferroni adjustment of significance levels when interpreting the results for single-type infections. No such adjustment was used for infections with any type, oncogenic and non-oncogenic types. This decision was based on the fact that in addition to pre-specifying these analyses, the multiple-type outcomes are inter-dependent and conventional correction for multiple comparisons would be overly conservative. Because having infection with multiple HPV types (defined as 2 or more HPV types) was not associated with a significantly increased risk of HIV acquisition compared to infection with a single HPV type, this variable was not included in further analyses. In order to ascertain whether cytologic effects of HPV accounted for any association between HPV infection and HIV acquisition, we examined whether abnormal cytology was predictive of HIV acquisition.

Both self-collected and clinician-collected swabs were used for defining HPV status. If results from both collection methods were available at the same visit, a positive result from either test contributed to the definition of a positive HPV test. Baseline HPV status for particular types or groups of types was defined using HPV tests from the study enrollment visit, and used as a fixed covariate in analyses. We defined a cumulative HPV status indicator of ever having tested positive for a type or group of types at baseline or during follow-up (up to and including the first HIV positive visit for HIV converters), and considered this as a fixed covariate in analyses. We also defined a status indicator of persistent or non-persistent HPV infection and these were considered as fixed covariates in analyses. A type-specific infection was defined as persistent if any 2 consecutive tests were positive for a specific HPV type, or if any of the following conditions were met (if positive tests were not consecutive): for participants with 2 to 6 tests, ≥2 tests were positive but not >3 consecutive negative tests; for participants with 7 to 9 tests, ≥3 tests were positive but not >3 consecutive negative tests; for participants with 10 tests, ≥4 tests were positive but not >3 consecutive negative tests; for participants with 11 or 12 tests, 4 tests were positive but not >4 consecutive negative tests. A type-specific HPV infection that did not meet the above criteria was considered non-persistent. Participants with no visits positive for type-specific HPV were considered uninfected. Participants with HPV data from only one study visit were excluded from this analysis.

The following measures of HPV status were also considered as time-dependent covariates: Women were defined as having a “concurrent HPV infection” if HPV testing was positive at the HIV acquisition visit. Women were defined as having a “recent HPV infection” if they were positive for HPV at any visit within 6 months up to and including the HIV acquisition visit. We also performed a “sensitivity analysis of recent HPV infection” of women positive for HPV at any visit within 6 months but *excluding* the HIV acquisition visit. A participant visit was excluded from the analysis if there were no HPV results for a particular visit (for “concurrent HPV infection”) or at any time in the preceding 6 months (for “recent HPV infection”), resulting in different numbers of women-visits contributing to different analyses. Observations for visits for women who acquired HIV were censored after the HIV acquisition visit.

Cox proportional hazards regression models were used to evaluate associations between HPV status and HIV acquisition. Because of the discrete nature of follow-up, the Efron correction for tied failure times was employed in parameter estimation. The validity of the proportional hazards assumption was evaluated via a test of interaction between HPV status indicators and duration of follow-up. HPV status variables (described above) were included as either fixed (for prevalent, cumulative or persistent/non-persistent infection) or time-dependent covariates (for concurrent or recent infection). Although the data were pooled from the 2 arms of a negative randomized trial, we included study arm as a variable in the analyses in order to control for a possible effect of the intervention. Separate models were fitted for different HPV types. Models controlled for potentially confounding variables, including baseline demographic characteristics and biological/clinical measures (listed in Table 1), and time dependent behavioral and biological measures. Time dependent measures reflected results from visit-specific interviews and clinical exams.

**Table 1:** Demographic, behavioral, and biological/clinical characteristics of participants at baseline (N = 2040).

Characteristic	Number of Women (%) or Mean (range)
**Demographic**	
Age	
24 years or younger	758 (37.2%)
25 to 34 years	937 (45.9%)
35 years or older	345 (16.9%)
**Behavioral**	
High risk partner: at least one indicator^1^	1,343 (65.9%)
High risk behavior: at least one indicator^2^	491 (24.1%)
Lifetime number of sex partners, mean (range)	1.3 (1–20)
Age at first sex, mean (range)	18.6 (10–28)
Regular sex partner circumcised	
No	1,393 (68.3%)
Yes	328 (16.1%)
Don't know	319 (15.6%)
Living together with regular sex partner	1,956 (95.9%)
Frequency of condom use in past 3 months	
Never	615 (30.1%)
Sometimes	876 (43.0%)
Always	548 (26.9%)
Biological/Clinical	
Normal cytology at baseline^3^	1,673 (82.0%)
Tested seropositive for HSV-2	1,034 (50.8%)
Tested positive for curable STI(s)^4^	140 (6.9%)

1 Indicators include: Having any sexual partners test positive for HIV, suspect or know that regular partner had other sex partners in the last 3 months, ever had vaginal sex when partner was under influence of drugs/alcohol in last 3 months, regular partner was away from home for 1 or more months.

2 Indicators include: Any exchange of sex for money/food/drugs/shelter, 2 or more sexual partners within last 3 months, ever had vaginal sex under influence of drugs/alcohol in last 3 months, ever used needle for injectable drug use, ever had anal sex.

3 The remainder had abnormal cytology (defined as atypical squamous cells of undetermined significance or worse), unsatisfactory tests, or missing results.

4 At least one positive test for *N gonorrhoeae*, *C trachomatis*, *Trichomoniasis vaginalis* or syphilis at screening or enrollment.

## Results

### Characteristics of the study population

Of 2089 HIV-negative female participants in MIRA who were offered participation in the HPV sub-study, 2040 women (97.6%) consented and 1918 (94%) of participants completed the study. The mean age of study participants at enrollment was 27 years (range 18–49 years), and the median follow-up period was 21 months (range 12–24 months). Detailed demographic characteristics of participants have been published elsewhere [15] and are summarized in Table 1. Briefly, 95.9% of women reported living with a regular partner/husband. The mean number of lifetime sexual partners was 1.3 (range 1–20). Infection with *Neisseria gonorrhoeae*, *Chlamydia trachomatis*, *Trichomonas vaginalis* or syphilis was identified in 6.9% of women at baseline, and 50.8% of women had serologic evidence of prior exposure to HSV-2. A total of 88 women acquired HIV during follow-up, for an HIV incidence of 2.7 per 100 woman-years (95% CI = 2.2, 3.4).

Characteristics of women with prevalent, incident and persistent HPV infections in this cohort have been described elsewhere [18]. Briefly, among women with baseline evaluable HPV samples (N = 1987), prevalence of infection with any HPV type was 24.5%. Specific HPV types with prevalence >1% were: 58 (5.0%), 16 (4.7%), 70 (2.4%), 18 (2.3%), 33 (2.0%), 53 (1.8%), 31 (1.3%), 52 (1.3%) and 61 (1.0%). Incidence of any new HPV type within 11–16 months after enrollment was 23.3%. HPV types with incidence >1% were: 58 (2.4%), 16 (2.1%), 70 (1.7%), 18 (1.3%), 53 (1.2%), 31 (1.2%), 33(1.1%), 61 (1.1%) and 52 (1.0%).

### Effect of prevalent HPV infection on risk of HIV acquisition

We first examined whether prevalent HPV infection (defined as HPV detection at baseline) was predictive of HIV acquisition. In regression models adjusted for baseline variables associated with HIV acquisition, being positive at baseline for any HPV type or for any oncogenic HPV type was marginally associated, and being positive for HPV type 58 or 70 was significantly associated with HIV acquisition during the study (Table 2).

**Table 2:** Prevalent HPV infection as a predictor of HIV acquisition.

HPV status	Unadjusted Hazard Ratio	95% Confidence Interval	p-value	Adjusted Hazard Ratio^1^	95% Confidence Interval	p-value
**Any HPV**	1.84	1.19–2.86	0.006	1.55	0.99–2.42	0.053
**Any non-oncogenic HPV** 2	1.52	0.74–3.15	0.27	1.22	0.58–2.55	0.595
**Any oncogenic HPV** 3	1.78	1.10–2.86	0.018	1.56	0.96–2.52	0.069
**HPV16**	1.34	0.54–3.30	0.524	1.27	0.51–3.14	0.604
**HPV18**	0.49	0.68–3.53	0.480	0.47	0.06–3.37	0.451
**HPV31**	1.87	0.46–7.60	0.381	1.92	0.47–7.87	0.366
**HPV33**	1.82	0.58–5.77	0.306	1.51	0.47–4.80	0.486
**HPV52**	0.97	0.13–6.94	0.973	0.86	0.12–6.23	0.882
**HPV53**	1.31	0.32–5.30	0.709	1.11	0.27–4.51	0.888
**HPV58**	2.58	1.34–5.00	0.005	2.13	1.09–4.15	0.026
**HPV61**	1.23	0.17–8.83	0.838	1.09	0.15–7.84	0.935
**HPV70**	3.08	1.25–7.60	0.015	2.68	1.08–6.66	0.033

1 Adjusted for the following baseline characteristics: high risk partner (as defined in Table 1 footnote), positive test for *N gonorrhoeae*, *C trachomatis*, *Trichomoniasis vaginalis* or syphilis, cohabiting with regular sexual partner, age 25–34 years or >35 years (compared to age ≤24 years), positive serology for HSV-2, no condom use within the prior 3 months (compared to sometimes or always using condoms), and regular partner not circumcised.

2 Non-oncogenic HPV types defined as types 6, 11, 32, 40, 54, 55, 61, 69, 70, 83, 84, “mix”, and untyped.

3 Oncogenic HPV types defined as 16, 18, 26, 31, 33, 35, 39, 45, 51, 52, 53, 56, 58, 59, 66, 68, 73 and 82v.

### Effect of HPV infection during study participation on risk of HIV acquisition

HPV status at baseline does not necessarily reflect HPV status at later time points, since prevalent HPV infections can clear spontaneously or persist, and incident infections with other HPV types can occur over time. We therefore examined whether having an HPV infection at any time during study participation (baseline or follow-up) was associated with an increased risk of HIV acquisition. For this analysis, each participant was defined as HPV uninfected (negative for type-specific HPV at *every* visit) or HPV infected (positive for type-specific HPV at *any* visit up to and including the HIV acquisition visit). In regression models adjusted for baseline variables associated with HIV acquisition (as defined in Table 2 footnote), the following HPV status indicators were independently associated with HIV acquisition: being positive at any time during study participation for any non-oncogenic HPV type (adjusted HR [aHR] = 1.66; 95% CI = 1.06–2.62), for HPV 31 (aHR = 2.23; 95% CI = 1.11–4.46) or for HPV 58 (aHR = 2.01; 95% CI = 1.18–3.41). ). No significant deviations from proportional hazards were observed.

### Effect of abnormal cytology and persistent or non-persistent HPV infection on risk of HIV acquisition

Since HPV infection can result in epithelial lesions that might increase susceptibility to HIV, we examined the association between cytology results (collected at enrollment) and HIV acquisition during the study; having an abnormal cytology result (atypical squamous cells of undetermined significance or a more severe result) was not significantly associated with an increased risk of HIV acquisition (HR = 1.38; 95% CI = 0.80, 2.34). Persistent type-specific HPV infection is a well-established risk factor for development of cervical intraepithelial neoplasia. Therefore, we explored the association between persistent HPV infections and HIV risk in study participants. We defined an HPV infection as being persistent or non-persistent based on the proportion of positive type-specific tests for each participant. In regression models adjusted for baseline variables, persistent infections had no effect on HIV risk, but non-persistent infection with either oncogenic or non-oncogenic HPV types were both significantly associated with an increased risk of HIV acquisition (Table 3). In analyses of type-specific HPV infections adjusted for baseline variables, persistent infections did not confer risk regardless of the HPV type whereas non-persistent infections with the following HPV types were significantly associated with an increased risk: HPV 31 (aHR = 3.03, 95% CI = 1.39, 6.58); HPV 53 (aHR = 2.35, 95% CI = 1.28, 4.94); and HPV 58 (aHR = 2.52, 95% CI = 1.28, 4.94).

**Table 3:** Persistent and non-persistent HPV infection as predictors of HIV acquisition.

HPV Status	Unadjusted HR	95% confidence interval	P value	Adjusted HR^1^	95% confidence interval	P value
**Any non-oncogenic HPV** 2 **:**						
**Persistent**	1.50	0.56–1.84	0.279	1.24	0.59–2.60	0.574
**Non-persistent**	2.42	1.26–3.25	<0.001	2.09	1.27–3.44	0.004
**Any oncogenic HPV** 3 **:**						
**Persistent**	1.01	0.72, 3.15	0.969	0.82	0.45–1.50	0.523
**Non-persistent**	2.02	1.47, 3.98	0.003	1.67	1.03–2.70	0.038

1 Adjusted for the following baseline characteristics: high risk partner (as defined in Table 1 footnote), positive test for *N gonorrhoeae*, *C trachomatis*, *Trichomoniasis vaginalis* or syphilis, cohabiting with sexual partner, age 25–34 years or >35 years (compared to age ≤24 years), and positive serology for HSV-2.

2 Non-oncogenic HPV types defined as types 6, 11, 32, 40, 54, 55, 61, 69, 70, 83, 84 (mixed and untyped infections are excluded from the analysis).

3 Oncogenic HPV types defined as 16, 18, 26, 31, 33, 35, 39, 45, 51, 52, 53, 56, 58, 59, 66, 68, 73 and 82v.

### Effect of concurrent HPV infection on risk of HIV acquisition

To assess the effect of HPV infection at the time of HIV acquisition, we analyzed the effect of concurrent HPV infection on the risk of HIV acquisition. In unadjusted analyses, HIV acquisition was significantly associated with having concurrent infection with any non-oncogenic HPV type, with any oncogenic HPV type, or with HPV type 31, 58 or 70 (Table 4). Given that risk factors for acquisition of HIV can also change over time, we controlled for time-dependent variables such as HSV-2 serology status, incident STIs, report of a new sexual partner and report of condom use at the last sexual encounter prior to the current visit, as well as baseline characteristics. HIV acquisition was independently associated with having concurrent infection with any non-oncogenic HPV type, with any oncogenic HPV type, or with HPV type 31 or 70 (Table 4). Significance of results for the single-type infections (31 and 70) was preserved at the 5% level after Bonferroni adjustment of p-values to account for 9 separate comparisons.

**Table 4:** Concurrent HPV infection as a predictor of HIV acquisition.

HPV Status	Unadjusted Hazard Ratio	95% confidence interval	p-value	Adjusted Hazard Ratio^1^	95% confidence interval	p-value
**Any HPV type**	2.25	1.45–3.75	<0.001	1.59	0.98–2.58	0.063
**Any non-oncogenic HPV** 2	2.37	1.45–3.83	<0.001	1.70	1.02–2.85	0.042
**Any oncogenic HPV** 3	2.65	1.65–4.26	<0.001	1.96	1.16–3.30	0.012
**HPV 16**	1.80	0.83–3.92	0.139	1.38	0.61–3.13	0.445
**HPV 18**	1.95	0.71–5.36	0.194	1.37	0.47–4.02	0.568
**HPV 31**	6.23	3.00–13.00	<0.001	4.25	1.81–9.97	0.001
**HPV 33**	2.13	0.78–5.83	0.142	1.34	0.49–3.88	0.537
**HPV 52**	0.84	0.12–6.02	0.859	0.73	1.00–5.45	0.759
**HPV 53**	2.43	0.89–6.65	0.085	2.21	0.78–6.24	0.133
**HPV 58**	2.82	1.40–5.66	0.004	1.82	0.83–4.00	0.135
**HPV 61**	0.56	0.77–4.01	0.561	0.46	0.06–3.37	0.448
**HPV 70**	3.53	1.62–7.69	0.001	3.30	1.50–7.29	0.003

1 Adjusted for Baseline variables: study arm, high risk behavior (as defined in Table 1 footnote), high risk partner (as defined in Table 1 footnote), positive test for *N gonorrhoeae*, *C trachomatis*, *Trichomoniasis vaginalis* or syphilis, cohabiting with sexual partner, age 25–34 years or >35 years (compared to age ≤24 years), positive serology for HSV-2, no condom use within the prior 3 months (compared to sometimes or always using condoms), and regular partner not circumcised and Time-dependent variables: condom non-use since last visit; new partner since last visit; circumcision status of new partner; incident infection since the last visit with HSV-2, *N gonorrhoeae*, *C trachomatis*, *Trichomoniasis vaginalis*.

2 Non-oncogenic types defined as types 6, 11, 32, 40, 54, 55, 61, 69, 70, 83, 84, “mix”, and untyped.

3 Oncogenic types defined as 16, 18, 26, 31, 33, 35, 39, 45, 51, 52, 53, 56, 58, 59, 66, 68, 73 and 82v.

### Effect of recent HPV infection on risk of HIV acquisition

In order to determine whether HPV status preceding the time of HIV acquisition increased the risk of HIV infection, we defined “recent HPV infection” as HPV status at any visit within the previous 6 months, including the current visit. Having a recent HPV infection with any HPV type or with any oncogenic HPV type was independently associated with an increased risk of HIV acquisition (Table 5). We conducted a sensitivity analysis to determine the effect of recent HPV infection within the past 6 months but *excluding* the current visit; having an HPV infection with any non-oncogenic or oncogenic HPV types was independently associated with an increased risk of HIV acquisition (Table 5).

**Table 5:** Recent HPV infection (within 6 months of HIV acquisition visit) as a predictor of HIV acquisition.

Infection with:	Including current visit Adjusted Hazards Ratio^1^	95% confidence interval	p- value	Excluding current visit Adjusted Hazards Ratio^1^	95% confidence interval	p-value
**Any HPV**	1.63	1.00–2.66	0.052	1.59	0.97–2.58	0.063
**Any Non-oncogenic HPV** 2	1.50	0.92–2.43	0.104	1.70	1.02–2.85	0.042
**Any Oncogenic HPV** 3	1.95	1.19–3.21	0.008	1.96	1.16–3.30	0.012

1 Adjusted for Baseline variables: study arm, high risk behavior (as defined in Table 1 footnote), high risk partner (as defined in Table 1 footnote), positive test for *N gonorrhoeae*, *C trachomatis*, *Trichomoniasis vaginalis* or syphilis, cohabiting with sexual partner, age 25–34 years or >35 years (compared to age ≤24 years), positive serology for HSV-2, no condom use within the prior 3 months (compared to sometimes or always using condoms), and regular partner not circumcised and Time-dependent variables: condom non-use since last visit; new partner since last visit; circumcision status of new partner; incident infection since the last visit with HSV-2, *N gonorrhoeae*, *C trachomatis*, *Trichomoniasis vaginalis*.

2 Non-oncogenic types defined as types 6, 11, 32, 40, 54, 55, 61, 69, 70, 83, 84, “mix”, and untyped.

3 Oncogenic types defined as 16, 18, 26, 31, 33, 35, 39, 45, 51, 52, 53, 56, 58, 59, 66, 68, 73 and 82v.

## Discussion

In this large cohort of Zimbabwean women, we found consistently increased risk of HIV acquisition associated with cervico-vaginal HPV infection, and specific HPV types (HPV 31, 58 and 70) were implicated in the association. These results suggest a previously unrecognized role of HPV in HIV acquisition among women. An obvious explanation for the observed association is that it is attributable to a partner with both infections, and we cannot definitively rule out the effect of sexual exposure to both infections as the explanation for the association. However, demographic information collected at enrollment and at each quarterly visit allowed us to control for time-dependent variables such as new sexual partners, HSV-2 serostatus, new STIs, or high-risk behavior by the participant or her regular partner. In addition, the results from our sensitivity analysis suggest that HPV infection preceded HIV acquisition.

Most HPV infections are cleared by innate and adaptive immune mechanisms [19], making it is biologically plausible that the local immune response elicited by HPV infection predisposes to HIV acquisition, and that women in the process of clearing an HPV infection might be at increased risk. This possibility is supported by our finding that non-persistent HPV infections were associated with increased risk of HIV acquisition whereas persistent infections were not. HPV infection does not cause epithelial ulcerative lesions but rather is associated with hyperproliferative changes such as warts, cervical intraepithelial neoplasia or cancer; these lesions are known to be infiltrated by HIV target cells such as lymphocytes and macrophages [20], . Persistent HPV infections are associated with an increased risk of developing precancerous lesions, but in our study were not associated with an increased risk of HIV acquisition. These results suggest that clearance of HPV infection rather than the dysplastic effect of HPV on epithelial cells confers HIV risk. We do not known whether the women in this study had HPV-associated lesions at the time of HIV acquisition, and although we found no significant association between abnormal cytology (which detects HPV-associated lesions) and risk of HIV infection, this result may have been limited by lack of power. Additional studies incorporating frequent cytological and colposcopic evaluations are required to definitively establish the role of HPV-related lesions in HIV transmission. The observed association between HPV infection and HIV acquisition might also be due to unmeasured confounding variables such as social networks; although we adjusted for multiple variables that measure risk of HIV through sexual activity, this report cannot definitively establish the exact role of HPV infection in risk of HIV acquisition.

Our finding of HPV type-specific associations (HPV 31, 58 and 70) raises the possibility that biological processes might underlie the association with HIV risk. HPV 70 is a non-oncogenic HPV type in clade A7 whereas HPV 31 and 58 are oncogenic HPV types in clade A9. HPV16, another member of clade A9, is the most common HPV type in the African continent [23]; it was second in prevalence after HPV 58 in the Zimbabwean women in our study (4.7%), but it did not confer significantly increased risk of HIV acquisition in any of our analyses. The reason for the HIV risk association with a subset of HPV types is unknown but may reflect type-specific differences in the host immune response to HPV. The relatively high prevalence of HPV 58 and HPV 70 in this cohort, and the observed association of these types with HIV acquisition, suggest that the contribution of HPV to HIV acquisition risk may vary in different populations depending on the distribution of HPV types and host genetic polymorphisms. The currently available HPV vaccines target HPV 6, 11, 16 and 18, which are not the HPV types identified with HIV risk in our study (HPV 31, 58 and 70). Further investigation into host and HPV type-specific differences in the immune/inflammatory responses to HPV infection will help to elucidate possible biological mechanisms by which HPV infection could contribute to HIV transmission.

The strengths of our study are the large sample size, the high retention rate, and the abundance of data about HPV status and risk factors collected at frequent intervals over time. A limitation of this study is that the commonly used definition of HPV infection cannot differentiate between the presence of biologically active HPV *versus* the detection of HPV DNA due to deposition in the genital tract during a recent sexual encounter. For this reason and due to the observational study design, these results cannot establish causation between HPV infection and HIV acquisition, but they suggest that HPV is a potentially significant risk factor for HIV acquisition in women. To date, little is known about the association between HPV and HIV transmission. In a recent study in men who have sex with men, anal infection with 2 or more HPV types significantly increased the risk of HIV infection (HR 3.5, 95% CI = 1.2–10.6) [12]. Given the high prevalence of anogenital HPV infection, further investigation of the role of HPV in HIV acquisition is warranted. A better understanding of the factors underlying the HPV-HIV association will improve our understanding of the pathogenesis of HIV transmission.
